# First Nation-Wide Analysis of Food Safety and Acceptability Data in Lebanon

**DOI:** 10.3390/foods9111717

**Published:** 2020-11-22

**Authors:** Samer Kharroubi, Nivin A. Nasser, Marwa Diab El-Harakeh, Abdallah Alhaj Sulaiman, Issmat I. Kassem

**Affiliations:** 1 Department of Nutrition and Food Sciences, Faculty of Agricultural and Food Sciences, American University of Beirut (AUB), Beirut 1107 2020, Lebanon; sk157@aub.edu.lb (S.K.); mhd26@mail.aub.edu (M.D.E.-H.); asa89@mail.aub.edu (A.A.S.); 2 Center for Food Safety, Department of Food Science and Technology, University of Georgia, 1109 Experiment Street, Griffin, GA 30223-1797, USA; nivin.nasser@uga.edu

**Keywords:** food safety, Lebanon, foodborne pathogens, foodborne diseases, *Salmonella*, *E. coli*, *S. aureus*, meat, poultry, dairy

## Abstract

The challenges to food safety in Lebanon are numerous and have coalesced to pose a serious public health concern. This is evident in well-documented food poisoning outbreaks and adulteration cases. In response, the Lebanese government initiated an unprecedented food safety campaign (2015–2017) that aimed to test food samples that were randomly collected from foodservices and industries across the country. The data were made available publicly, but they were never analyzed to prioritize and determine high risk foods and most prevalent contaminants nationally or across governorates. To answer these questions, we performed an in-depth statistical analysis of the data, which included 11,625 individual food samples. Our analysis showed that water (55% of tested water samples), spices (49.3%), red meat (34.4%), poultry (30.9%) and dairy (28.3%) were the main foods associated with the highest rejection rates. The most common biological contaminants detected in rejected foods were sulfate-reducing bacteria (34.7%), *Escherichia coli* (32.1%), coliforms (19.6%), *Staphylococcus aureus* (12.8%), and *Salmonella* (11.6%). We conclude that Lebanon needs rigorous and sustainable programs to monitor the quality and safety of foods. Given the lack of resources, we recommend putting emphasis on extensive outreach programs that aim at enhancing food safety knowledge from farm to fork.

## 1. Introduction

Access to sufficient quantities of safe and nutritious food is a prerequisite for sustaining life and good health. However, chemical and microbiological contamination of food can lead to a plethora of human diseases that range from diarrhea to cancer [1,2]. The majority of foodborne diseases have been attributed to microbial pathogens, such as *Escherichia coli*, norovirus, *Campylobacter* and non-typhoidal *Salmonella* [3]. Furthermore, it is well documented that these foodborne pathogens exert a significant toll on public health and economies in both developed and developing countries [4]. Global estimates show that 600 million individuals (~1 in 10 people) are affected by foodborne illnesses every year, which leads to the death of 420,000 individuals [3]. Subsequently, outbreaks of foodborne illnesses adversely impact health care systems as well as national food brands, trade, tourism, and socioeconomic growth, especially in developing countries [5]. In these countries, foodborne diseases perpetuate the vicious cycle of disease, malnutrition, and poverty, which significantly affect the most vulnerable populations, including infants, young children, the elderly, and nutritionally deprived or immunocompromised individuals [5]. In addition, children under the age of 5 years are at a notably high risk, comprising almost a third (125,000) of the total foodborne disease-associated deaths per year [3]. Furthermore, the World Bank estimated the yearly cost of treating foodborne illnesses at $15 billion and total productivity loss in low- and middle-income countries at $95.2 billion [6]. Despite the impact of foodborne diseases on public health and economy, many developing countries lack sustainable food safety systems and lag in monitoring and controlling food contaminants [7]. The latter applies to Lebanon, a relatively small Mediterranean country, which is currently experiencing significant challenges in its food safety and security systems. Lebanon is located in the Eastern Mediterranean which ranked third among the regions with the highest estimated burden of foodborne diseases per population [3].

The food industry in Lebanon encompasses 18.2% of all factories and 25% of the total workforce [8]. Food products are the number one exports in Lebanon, accounting for ~$1.7 billion in revenue [8]. However, Lebanon is currently struggling with wide-spread pollution, weakened infrastructure, political and civil unrest, and the most significant and debilitating economic crisis in its recent history [9,10,11,12]. Consequently, food safety and security issues in Lebanon have been on the rise with various cases of spoiled and expired food, fraud, and outbreaks that have received national attention [9]. Furthermore, in a study conducted between 2011 and 2012 in Lebanon, *Salmonella* in chicken and meat was considered a major source of foodborne infections [13], but the study focused only on *Salmonella* and did not include other foodborne pathogens. However, there are a few and sporadic studies that reported the presence of pathogens such as *Listeria monocytogenes*, *Brucella*, *Salmonella* and *E. coli* in a variety of foods in Lebanon [14]. Additionally, it is known that some exported Lebanese food items were found to be microbially contaminated. For example, *Salmonella* Montevideo was isolated from Lebanese sesame-seed based products imported into Australia and New Zealand [15]. Specifically, sesame-seed paste (tahini) was implicated in outbreaks of foodborne disease in Victoria (Australia) and Auckland (New Zealand) [15]. Furthermore, *S.* Bovismorbificans associated with tahini was implicated in a multistate outbreak in the USA [16]. Similarly, in 2020, the Canadian Food Inspection Agency recalled tahini because of possible contamination with *Salmonella* [17]. Taken together, these reports have raised concerns among consumers and the food industry in Lebanon.

Currently, there are a plethora of factors that contribute to the status of food safety in Lebanon, including (1) absence of national baseline data on chemical and microbiological food contaminants, (2) insufficient scientific and technical expertise in the field, (3) scant financial support, (4) poor foodborne illness/outbreak surveillance, (5) inconsistent governmental oversight, transparency, and implementation and enforcement of policies and laws, (6) overlapping and conflicting jurisdictions of governmental agencies that oversee food safety, and (7) weak awareness of safety procedures in small food businesses [9]. Furthermore, there is limited support for academic research on the prevalence and properties of food contaminants and subsequent control mechanisms [9,10,11,12,13,14,15,16,17,18,19,20,21,22,23,24,25,26,27,28,29,30,31,32]. Taken together, these issues have left their mark on both the national economy and public health [33]. The fundamental importance of the food industry to Lebanon necessitates the enhancement of national food safety, which could benefit from the establishment of sustainable systems for monitoring food contaminants and foodborne disease.

Amidst the growing concerns about food safety in Lebanon, the Lebanese Ministry of Public Health (MoPH) launched a campaign in 2015 for assessing the acceptability of food in the Lebanese markets [34]. Unannounced food safety inspections were performed and various samples of food, including water and raw ingredients, were randomly collected from restaurants, grocery stores, supermarkets, and small retail stores, as well as food production facilities [34]. The samples were evaluated for microbial contamination and results were periodically published on the official website of the MoPH [34]. This continued until July 2017, when resources and political drive waned and the campaign halted [34]. Although the sampling effort and scope of microbiological analysis were limited, the campaign was one-of-a-kind in Lebanon and should have resulted in crucial insights into national food safety. However, to our knowledge, there was no attempt to analyze the collected data in order to identify the most common microbial contaminants and/or the foods that pose the most risk to the Lebanese consumers. This analysis would have been crucial to inform policy, raise consumer awareness, and provide plans to focus limited resources to address food safety priorities. Consequently, the aim of our study was to analyze the data collected during the MoPH campaign between 2015–2017 in order to better understand microbial food safety, identify the most frequently contaminated foods and contaminants, and assess the risk associated with food in the Lebanese market.

## 2. Materials and Methods

### 2.1. Data Description

The data were collected during a campaign launched by the Lebanese MoPH from 2015 to 2017. The campaign involved random and uninformed inspections of various facilities across Lebanon, which included restaurants, grocery stores and supermarkets, butcheries, fisheries, roasteries, and bakeries. The data were shared publicly in 110 separate reports on the MoPH website (https://www.moph.gov.lb/en/Pages/4/126/food-safety) [34]. The reports were qualitative and included a description of the sample (food/drink/ingredient type), sample location, sampled foodservice or food-production facility, identity of detected contaminant(s), and the food unacceptability according to the criteria adopted by the Lebanese Standards Institution (LIBNOR) [34]. However, the reports did not always explicitly specify whether the food was cooked, raw, and/or ready-to-eat [34]. The last report was posted on 17 July 2017 [34].

A total of 11,625 samples were collected, including (1) raw, frozen, marinated and cooked meat (red meat, poultry meat, and fish), (2) water, (3) raw and cooked pastry and bread, (4) ready-to-eat foods, (5) dairy products, including yogurt, fermented yogurt and various types of cheese, (6) spices, (7) vegetables, and (8) raw and roasted nuts and dried fruits among others.

### 2.2. Statistical Analysis

Due to the diversity of the tested samples (cooked, raw, frozen, fresh, etc.), we categorized the samples based on food type to facilitate statistical analysis. Therefore, the categories analyzed were red meat, poultry meat, dairy, bakery, fish, nuts, desserts, spices, water, and other (which included various items such as vinegar, beans, jam, vegetables, coffee, and tea among others). The data in the published reports were then checked for completeness and were coded and logged into the Statistical Package for Social Sciences (SPSS Inc., Chicago, IL, USA) software version 18 for Windows, which was later used for statistical analyses. Descriptive statistics were performed and presented as frequencies and percentages for categorical variables. In addition, the associations between food category and type of microbiological contaminant as well as the prevalence of unacceptable (rejected) food categories were assessed using Chi-square test. Comparisons between governorates (the administrative divisions of Lebanon) were also performed using Pearson’s Chi-square test. Additionally, the association between the microbial acceptability of food categories and location of sampling (governorates) was explored using simple logistic regression, with microbiological acceptability as the dependent variable. To evaluate the correlates of microbial acceptability risk, a multiple logistic regression model was utilized. In this model, variables were included if they were significantly associated with the dependent variable in the univariate analysis. Odds ratios (OR) and their respective 95% confidence intervals were computed. All reported *p*-values were based on two-sided tests and were compared with a significance level of 5%.

## 3. Results

Analysis of the data from 2015–2017 (*n* = 11,625) showed that 71.3% (*n* = 8291) of all tested samples were labelled as microbially acceptable, whereas 28.7% (*n* = 3334) of the samples were unacceptable (Table 1). Based on the number of unacceptable samples within each food category, five food categories (red meat, poultry meat, dairy, spices, and water) were identified as high risk. These categories also contained the highest percentages of unacceptable samples per category, where water, spices, red meat, poultry meat, and dairy harbored 55.0%, 49.3%, 34.4%, 30.9%, and 28.3% of unacceptable samples per category, respectively (Table 1).

While assessing the distribution of unacceptable samples according to the governorate (geographical location), it was observed that 31.7% of all samples from the North governorate of Lebanon were rejected, while 29.9% and 27.3% of the samples were unacceptable in South Lebanon and Mount Lebanon, respectively (Table 2). Beirut, the capital of Lebanon, had the lowest percentage (23.8%) of unacceptable samples (Table 2). Statistical analysis revealed a significant variation in the prevalence of unacceptable food samples across governorates (*p*-value < 0.001; Table 2). Furthermore, odds ratio analysis indicated that it was almost 1.4 times more likely to encounter unacceptable food products in the North and South governorates compared to the governorate with the lowest food rejection percentage (Beirut) (Table 2). Further analysis of the data revealed that the food categories with the highest percentages of unacceptable samples included red meat, poultry meat, dairy, and spices in the North, South, Mount Lebanon, and the Bekaa governorates (Table 3). However, red meat, spices, and other foods were the three highest risk categories in the Beirut governorate (Table 3).

Evaluation of the microbial criterion that led to the rejection (unacceptability) of the food samples showed that sulfate-reducing bacteria (SRB), *E. coli*, aerobic bacteria, and coliforms were the most common contaminates (Table 4). The levels of these bacteria were the criteria for rejecting 34.7%, 32.1%, 21.7%, and 19.6% of the unacceptable samples, respectively (Table 4). In addition, unacceptable levels of *Staphylococcus aureus, Salmonella* spp., and yeast and fungi resulted in the rejection of 11.6%–12.8% of the samples (Table 4). Notably, red meat samples constituted 65.2% of all rejected samples contaminated with unacceptable levels of *S. aureus*, 46.2% with SRB, 55.3% with *E. coli*, 52.5% with *L. monocytogenes*, and 32.6% with *Salmonella* spp., respectively (Table 4). Additionally, poultry meat samples constituted 62.0% of all rejected samples contaminated with unacceptable levels of *Salmonella* spp., 33.8% with SRB, 23.2% with aerobic bacteria, and 22% with *L. monocytogenes*, respectively (Table 4). Dairy samples constituted 38.7% of all rejected samples contaminated with unacceptable levels of coliforms, 37.1% with yeast/fungi, 23.7% with *L. monocytogenes*, and 19.7% with *E. coli*, respectively (Table 4). A notable finding was that spices constituted 74.1% and 35% of the rejected samples due to aflatoxins and yeast/fungi, respectively (Table 4). Further analysis revealed that there was a statistically significant association between food group and the microbial contaminants (*p*-value < 0.001) (Table 4).

Although the MoPH reports did not always explicitly designate whether the tested food was ready-to-eat, screening the data showed that *L. monocytogenes* occurred in 14 cheese samples and 13 minced red meat samples (with or without herbs and onions), which are traditionally consumed without further cooking in Lebanon. Furthermore, *Salmonella* spp. were detected in six cheese and 54 minced red meat samples. Unacceptable levels of fecal indicators, *E. coli* and coliforms, occurred in 319 cheese samples, while *E. coli* was detected in 306 minced red meat samples. Additionally, the occurrence of a variety of microorganisms and toxins in spices demonstrates the contamination of ingredients used in different foods (Table 4).

## 4. Discussion

In response to increasing public concern, the ministry of public health (MoPH) in Lebanon launched a food safety campaign (2015–2017) [34]. Although the number of collected samples was modest and testing focused on a limited list of microbial contaminants, the campaign presented a chance to shed light on food safety and major foodborne contaminants in Lebanon using experimental data. Here, we downloaded the data from the public website of MoPH and performed in-depth statistical analysis. Our overarching objective was to provide awareness to stakeholders on food safety as well as prioritize the risks for efficient allocation of resources and control efforts.

It was notable that 28.7% of all tested samples were found to be microbially unacceptable (Table 1). Accordingly, these samples were rejected by the MoPH, because they pose a potential food safety and quality risk. The number of rejected samples showed that more than one in four of the tested samples were contaminated. This is important, because food contaminated with pathogens like *Salmonella* and *Listeria* and toxins secreted by fungi (aflatoxins), *S. aureus*, and *C. botulinum* is known to cause life-threatening infections and acute and chronic illnesses in humans [35,36,37,38,39]. This includes diarrhea, nausea, vomiting, stomach cramps, sepsis, renal failure, meningitis, respiratory and muscular paralysis, miscarriage, stillbirth, and cancer among others [35,36,37,38,39]. Additionally, SRB are associated with ulcerative colitis and are thought to trigger inflammatory bowel diseases (IBD) [40]. However, when considering the microbial parameters (Table 4), it can be immediately observed that the testing was not comprehensive. For example, foodborne viruses and parasites, such as norovirus, hepatitis A, and *Cryptosporidium*, were not tested. This is important, because norovirus is the leading cause of foodborne gastroenteritis worldwide [41]. Additionally, 10,400 cases of hepatitis A were reported in Lebanon between 2005 and 2017 [42]. Similarly, there are recent reports of *Cryptosporidium* and other parasitic infections in the Lebanese population [43,44,45]. The MoPH reports did not also include testing of potential chemical contaminants in the food. Taken together, this suggests that there is a possibility that the rejection rate might increase if other microbial pathogens/indicators/chemicals were to be tested. Consequently, the Lebanese consumers might be at a notably high risk of contracting foodborne diseases, which has a significant impact on public health and highlights the pre-emptory need to address food safety in Lebanon.

When analyzing the distribution of unacceptable food samples across governates, we noted that the highest number (31.7%) of rejected samples were collected in the North governate, while the lowest (23.8%) was in Beirut, the capital of Lebanon (Table 2). This perhaps reflected a possible socioeconomic impact on food safety in Lebanon, because the North is generally known to be facing more challenges in poverty, income distribution, and infrastructure in comparison to the rest of the country [46,47]. However, red meat, poultry meat, dairy, and spices samples were the most problematic in every governorate (Table 3). Regardless, the high rate of microbially unacceptable samples in essential food categories highlights the potential risks of food contamination to the Lebanese population, especially in the poorest governorates.

Several important foodborne pathogenic bacteria, such as *Salmonella*, *L. monocytogenes*, *S. aureus*, and *C. botulinum*, were detected in the tested food samples in Lebanon (Table 4). A notable observation was also the presence of aflatoxins in spices (Table 4). Therefore, the data highlight gaps in the hygienic and sanitation systems deployed in the food chain in Lebanon. For example, indicators of fecal contamination (*E. coli* and coliforms) and poor sanitation (*S. aureus*) [48] were among the major microbial contaminants in rejected food samples (Table 4). The presence of the aforementioned pathogens and toxins in foods might have severe health repercussions, especially in children, the elderly, and immunocompromised and disenfranchised populations. Given the status of the economy and the current increase in the cost of medical care in Lebanon, these food contaminants might inflict higher mortalities and morbidities in susceptible populations. This scenario is even more plausible because of the proliferation of antimicrobial resistance in food systems in Lebanon [14,21,22,28,29,32,49], which can lead to recalcitrant and hard-to-treat infections [50].

Red meat, poultry, and dairy were among the most contaminated food categories in Lebanon. The contamination of red meat is notable, because it can be consumed raw as part of popular Lebanese dishes, which increases the risk to consumers. Poultry and dairy are also popular in the Lebanese diet and all three food categories constitute an essential component of the economy [51,52,53]. Here, it should be noted that the observed results in this study largely depend on the methodologies deployed by the MoPH personnel, their skills, and budget. Regardless, food safety initiatives in Lebanon might benefit from focusing on these food categories to identify and mitigate contamination effectively. In turn, this will also considerably reduce the burden of foodborne infections in Lebanon and allow food safety stakeholders to operate sustainably.

Lebanon lacks a well-established food safety infrastructure to aid in identifying and tracking foodborne outbreaks [33]. Therefore, the burden of foodborne diseases in Lebanon is underestimated [9]. This, along with deficient resources and infrastructure, will affect the actions and policies employed to control foodborne disease outbreaks [54]. However, based on our analysis, it can be readily concluded that food safety must be a central concern for Lebanon, because evidence points to widespread contamination of foods that can have detrimental effect on health and economy. This is predicted to become more severe, because of the current political and economic crises in Lebanon. Our analysis can help stakeholders to focus and prioritize their engagement in food safety in Lebanon by addressing the foods that pose the highest risk.

Interventions which, if implemented, would substantially improve food safety in Lebanon must include (1) prioritizing the evaluation of the major food systems and the anecdotal sources of foodborne contamination (local and imported), (2) establishing a baseline for a national surveillance system for foodborne pathogens (bacteria, viruses and parasites) and antimicrobial resistance, (3) recruiting competent staff and using computerized systems and upgraded laboratories, (4) defining roles and tasks of various official and private agencies involved in food safety, (5) educating, training and raising awareness among the public and business owners, and (6) devising control measures to reduce the risk for consumers.

## Figures and Tables

**Table 1:** The number of samples in each food category that were found to be microbially unacceptable or acceptable based on the reports of the Ministry of Public Health (MoPH) in Lebanon. Samples were rejected due to spoilage and/or because they were deemed to pose a food safety risk to consumers.

Food Categories	Unacceptable	Acceptable	Total	*p*-Value ^b^
	N (% ^a^)	N (%)	N (%)	
Red Meat	1132 (34.4)	2154 (65.5)	3286 (100)	< 0.001
Poultry Meat	760 (30.9)	1698 (69.1)	2458 (100)	
Dairy	530 (28.3)	1343 (71.7)	1873 (100)	
Bakery	52 (11.2)	413 (88.8)	465 (100)	
Fish	51 (20.8)	194 (79.2)	245 (100)	
Nuts	40 (10.8)	330 (89.2)	370 (100)	
Desserts	167 (22.9)	561 (77.1)	728 (100)	
Spices	387 (49.3)	398 (50.7)	785 (100)	
Water	94 (55.0)	77 (45.0)	171 (100)	
Other	121 (9.7)	1123 (90.3)	1244 (100)	
Total	3334 (28.7)	8291 (71.3)	11,625 (100)	

^a^ Percentages were calculated by dividing the number of unacceptable/acceptable samples in each food category to the total number of samples in the same food category. ^b^ *p*-value was derived from Chi-square test that was conducted to evaluate the association between food categories and microbial acceptability of food samples (unacceptable vs. acceptable).

**Table 2:** The distribution of total food samples that were found to be microbially unacceptable or acceptable by the Ministry of Public Health (MoPH) across Lebanese governorates (administrative divisions of Lebanon).

Governorate	Unacceptable	Acceptable	Significance of Differences between Governorates	Odds Ratio (95% CI); *p*-Value ^c^
	N (% ^a^)	N (%)		
North	1121 (31.7)	2413 (68.3)	χ^2^ = 39.73, *p*-value ^b^ < 0.001	1.48 (1.13, 1.95); *p* = 0.005
Mount Lebanon	901 (27.3)	2403 (72.7)		1.20 (0.91, 1.58)
South	767 (29.9)	1799 (70.1)		1.36 (1.03, 1.80); *p* < 0.029
Bekaa	473 (24.72)	1446 (75.3)		1.05 (0.79, 1.39)
Beirut	72 (23.8)	230 (76.2)		1.0
Total no. of samples (%)	3334 (28.7)	8291 (71.3)		

^a^ Percentages were calculated by dividing the number of unacceptable/acceptable samples in each governorate to the total number of collected samples in the same governorate. ^b^ *p*-value was derived from Chi-square test that was conducted to compare differences in microbial acceptability of food samples (unacceptable vs. acceptable) by governorates in Lebanon. ^c^ The strength of association between the microbial acceptability of food and location of sampling (governorates) was explored using simple logistic regression, with microbial acceptability as the dependent variable. Odds ratios (OR) and their respective 95% confidence intervals (CI) were computed and *p*-values were also derived to test significance of differences between Beirut and the other governorates.

**Table 3:** Comparison of the number of microbially unacceptable samples in each food category across Lebanese governorates (administrative divisions of Lebanon).

Governorate	Red Meat N = 1132 (%)	Poultry Meat N = 760 (%)	Dairy N = 530 (%)	Bakery N = 52 (%)	Fish N = 51 (%)	Nuts N = 40 (%)	Desserts N = 167 (%)	Spices N = 387 (%)	Water N = 94 (%)	Other N = 121 (%)	Total
North N (%)	439 (39.2)	290 (25.9)	184 (16.4)	4 (0.4)	8 (0.7)	14 (1.2)	44 (3.9)	88 (7.9)	12 (1.1)	38 (3.4)	1121
Mount Lebanon N (%)	324 (36.0)	231 (25.6)	80 (8.9)	8 (0.9)	20 (2.2)	12 (1.3)	63 (7.0)	98 (10.9)	30 (3.3)	35 (3.9)	901
South N (%)	230 (30.0)	144 (18.8)	156 (20.3)	31 (4.0)	12 (1.6)	8 (1.0)	31 (4.0)	108 (14.1)	36 (4.7)	11 (1.4)	767
Bekaa N (%)	117 (24.7)	88 (18.6)	106 (22.4)	8 (1.7)	4 (0.8)	6 (1.3)	25 (5.3)	85 (18.0)	14 (3.0)	20 (4.2)	473
Beirut N (%)	22 (30.6)	7 (9.7)	4 (5.6)	1 (1.4)	7 (9.7)	0 (0.0)	4 (5.6)	8 (11.1)	2 (2.8)	17 (23.6)	72

Percentages were calculated by dividing the number of unacceptable samples for each food category to the total number of unacceptable samples in each region.

**Table 4:** The distribution of microbial contaminants that resulted in the rejection of samples across food category based on the reports of the Ministry of Public Health (MoPH) in Lebanon.

	Food Categories											
Microbial Contaminant	Red Meat N = 1132 (% ^a^)	Poultry Meat N = 760 (%)	Dairy N = 530 (%)	Bakery N = 52 (%)	Fish N = 51 (%)	Nuts N = 40 (%)	Desserts N = 167 (%)	Spices N = 387 (%)	Water N = 94 (%)	Other N = 121 (%)	Total N (% ^b^) for Each Contaminant	*p*-Value ^c^
Sulfate-reducing bacteria (SRB)	534 (46.2)	391 (33.8)	8 (0.7)	5 (0.4)	11 (0.95)	11 (0.95)	3 (0.3)	159 (13.8)	27 (2.3)	7 (0.6)	1156 (34.7)	< 0.001
*E. coli*	592 (55.3)	200 (18.7)	211 (19.7)	7 (0.7)	8 (0.7)	4 (0.4)	17 (1.6)	8 (0.7)	10 (0.9)	14 (1.3)	1071 (32.1)	< 0.001
Aerobic bacteria	190 (26.3)	168 (23.2)	57 (7.9)	26 (3.6)	31 (4.3)	-	58 (8.0)	82 (11.3)	69 (9.5)	42 (5.8)	723 (21.7)	< 0.001
Coliforms	26 (4.0)	9 (1.4)	253 (38.7)	16 (2.4)	1 (0.2)	6 (0.9)	115 (17.6)	99 (15.1)	71(10.9)	58 (8.87)	654 (19.6)	< 0.001
*S. aureus*	279 (65.2)	43 (10.0)	67 (15.7)	3 (0.7)	8 (1.9)	-	18 (4.2)	3 (0.7)	1 (0.2)	6 (1.4)	428 (12.8)	< 0.001
*Salmonella* spp.	126 (32.6)	240 (62.0)	6 (1.6)	-	1 (0.3)	-	5 (1.3)	2 (0.5)	-	7 (1.8)	387 (11.6)	< 0.001
*Streptococcus* spp.	7 (8.9)	3 (3.8)	5 (6.3)	-	2 (2.5)	-	4 (5.1)	14 (17.7)	39 (49.4)	5 (6.3)	79 (2.4)	< 0.001
*Listeria monocytogenes*	31 (52.5)	13 (22.0)	14 (23.7)	1 (1.7)	-	-	-	-	-	-	59 (1.8)	< 0.001
*Pseudomonas aeruginosa*	-	-	-	-	-	-	-	-	48 (100.0)	-	48 (1.4)	< 0.001
*Clostridium botulinum*	4 (18.2)	2 (9.1)	1(4.5)	-	1 (4.5)	-	1(4.5)	2(9.1)	1(4.5)	10 (45.5)	22 (0.7)	< 0.001
Yeast/fungi	11 (2.6)	8 (1.9)	160 (37.1)	24 (5.6)	3 (0.7)	17 (3.9)	20 (4.6)	151 (35.0)	-	37 (8.6)	431 (12.9)	< 0.001
Aflatoxin	1 (3.7)	-	-	-	-	5 (18.5)	-	20 (74.1)	-	1 (3.7)	27 (0.8)	< 0.001

^a^ Percentages were calculated by dividing the number of samples rejected for each microbial contaminant within each food category to the total number of samples rejected for the same microbial contaminant ^b^ Total percentages were calculated by dividing the number of samples rejected for each microbial contaminant to the total number of rejected samples. ^c^ *p*-values were derived from Chi-square tests that were conducted to examine the associations between food categories and each type of microbial contaminant (Yes vs. No).
